# Altered behavioral and amygdala habituation in high-functioning adults with autism spectrum disorder: an fMRI study

**DOI:** 10.1038/s41598-017-14097-2

**Published:** 2017-10-19

**Authors:** Friederike I. Tam, Joseph A. King, Daniel Geisler, Franziska M. Korb, Juliane Sareng, Franziska Ritschel, Julius Steding, Katja U. Albertowski, Veit Roessner, Stefan Ehrlich

**Affiliations:** 1 Division of Psychological and Social Medicine and Developmental Neurosciences, Faculty of Medicine, Technische Universität Dresden, Fetscherstraße 74, 01307 Dresden, Germany; 2 Translational Developmental Neuroscience Section, Eating Disorder Research and Treatment Center at the Department of Child and Adolescent Psychiatry, Faculty of Medicine, Technische Universität Dresden, Fetscherstraße 74, 01307 Dresden, Germany; 3 Department of Child and Adolescent Psychiatry, Faculty of Medicine, University Hospital C. G. Carus, Technische Universität Dresden, Fetscherstraße 74, 01307 Dresden, Germany; 4 Department of Psychology, Technische Universität Dresden, Zellescher Weg 17, 01069 Dresden, Germany

## Abstract

Habituation to repeatedly presented stimuli is an important adaptive property of the nervous system. Autism spectrum disorder (ASD) has been associated with reduced neural habituation, for example in the amygdala, which may be related to social impairments. The main focus of this study was to investigate habituation effects on the level of behavioral responses as well as amygdala responses in adults with ASD during a working memory task flanked by task-irrelevant face stimuli. Twenty-two patients with high-functioning autism and 24 healthy controls (HC) were included in this functional magnetic resonance imaging (fMRI) study. We employed an established habituation index to investigate habituation effects. Suggestive of altered habituation, the habituation index showed a decrement of reaction time over the course of the experiment in the HC but not in the ASD group. Similarly, an expected pattern of habituation was evident in amygdala activation in HC but absent in ASD participants. These results provide evidence that habituation may be altered not only on a neural, but also on a behavioral level in ASD. While more research is needed to develop a better understanding of the underlying mechanisms, the current findings support the possibility that deficient habituation may be a biomarker of ASD.

## Introduction

Autism spectrum disorder (ASD) is a pervasive developmental disorder characterized by persistent deficits in social communication and interaction and restricted, repetitive patterns of behavior, interests, or activities. Symptoms first emerge in early development and cause significant impairment in social, occupational, or other important areas of functioning^1^. Though not part of the diagnostic criteria, other symptoms including abnormal face processing have been identified in individuals with ASD^2,3^. It has been suggested that amygdala dysfunction may play a central role in deficits in social behavior in ASD^4^. There is strong evidence for a critical function of the amygdala in emotional processing and detection of innate, biologically and socially relevant information^5,6^. This is supported by the amygdala’s stronger activation to faces than to other visual stimuli^6,7^, especially when perceiving and recognizing emotions in facial expressions^8^. The underlying mechanisms for amygdala abnormalities in ASD are not yet fully understood. Different explanations have been proposed, i.e. an early developmental failure involving the amygdala with a cascading influence on the development of other cortical areas that mediate social perception^9^ or an impact of genotype^10^.

While several studies have investigated amygdala activation magnitude to face stimuli in ASD with mixed results^11–16^, the temporal dynamics of amygdala activation may be even more meaningful than activation magnitude^3,17,18^. One definition for habituation is a decrease in response strength^19^ as well as other parameters of response, f. ex. frequency or duration of response^20^, to repeatedly presented stimuli. Habituation is an important adaptive property of the nervous system improving selective attention and response to salient environmental aspects by ignoring familiar, inconsequential stimuli^20,21^. The human amygdala habituates to diverse stimuli including faces^17,22,23^. Several studies have delivered insight into amygdala habituation in ASD and have provided evidence for altered temporal dynamics of amygdala activation in ASD^3,10,24,25^. All but one of these studies displayed faces as task-relevant stimuli^3,10,24^ which might have affected habituation as some researchers view it as a decrement of response to irrelevant and inconsequential stimuli^20,21^. Furthermore, there are still only very few studies investigating amygdala habituation in adult ASD subjects^3,25^.

To gain a broader understanding of the underlying mechanisms of abnormalities in emotional processing and social impairment in ASD, the current study investigated amygdala habituation to face stimuli in a sample of high-functioning adults with ASD. To this end, we analyzed behavioral and functional magnetic resonance imaging (fMRI) data acquired during an adapted emotional face n-back task similar to that introduced by Ladouceur *et al*.^26^. Our experimental working memory task displayed faces as distractors, thereby encouraging selective attention to target stimuli and thus possibly enhancing habituation to the distractors. Addressing the need to use a standardized measure of habituation, we adopted the approach previously employed by Plichta *et al*.^17,27^. This approach has been shown to reliably quantify the absolute degree of habituation independent of the initial reactivity within-subjects during an emotional face task. We predicted that habituation would be reduced or absent in ASD as gauged by both behavioral performance and amygdala activation.

## Methods

### Participants

Twenty-five adults with high-functioning ASD and 25 healthy controls (HC), matched for age and gender, completed the fMRI protocol. All protocols received ethical approval by the institutional review board (IRB) of the TU Dresden (registered at the Office for Human Research Protections, IRB00001473, IORG0001076, study number: EK 14012011) and were carried out in accordance with the relevant guidelines and regulations. All participants gave written informed consent. Inclusion criteria for study participation were age of 18 years or above, an intelligence quotient of at least 85 and, for ASD participants, the established diagnosis of an autism spectrum disorder. HC participants were excluded if they had any history of psychiatric illness. We applied additional exclusion criteria for both groups. Complete details regarding exclusion criteria and comorbidities are provided in Supplementary Methods. Data for three participants with ASD and one HC were excluded due to an IQ below 85 (2 ASD), chance level performance (1 ASD) and excessive motion during the scan (1 HC). Overall, data from 22 individuals with ASD (19 male, 3 female, 21–55 years old) and 24 HC (19 male, 5 female, 23–56 years old) were included in the analyses. ASD participants were identified through a large specialized autism outpatient clinic and local self-help groups. Study data were collected and managed using the secure, web-based electronic data capture tool REDCap (Research Electronic Data Capture^28^).

### Clinical Measures

ASD diagnoses were confirmed with the ADOS (Autism Diagnostic Observation Schedule) for all ASD participants. Furthermore, autism symptoms were evaluated using the self-assessment questionnaires autism-spectrum quotient (AQ^29^), empathy quotient (EQ^30^) and systemizing quotient (SQ^31^). Intelligence quotient (IQ) was measured with the German adaptation of the Wechsler Adult Intelligence Scale^32^ for all ASD participants (retest if former test dated back to 4 years or more) and a short version of the Wechsler Adult Intelligence Scale for HC^33^.

### Experimental paradigm

**Figure 1:**
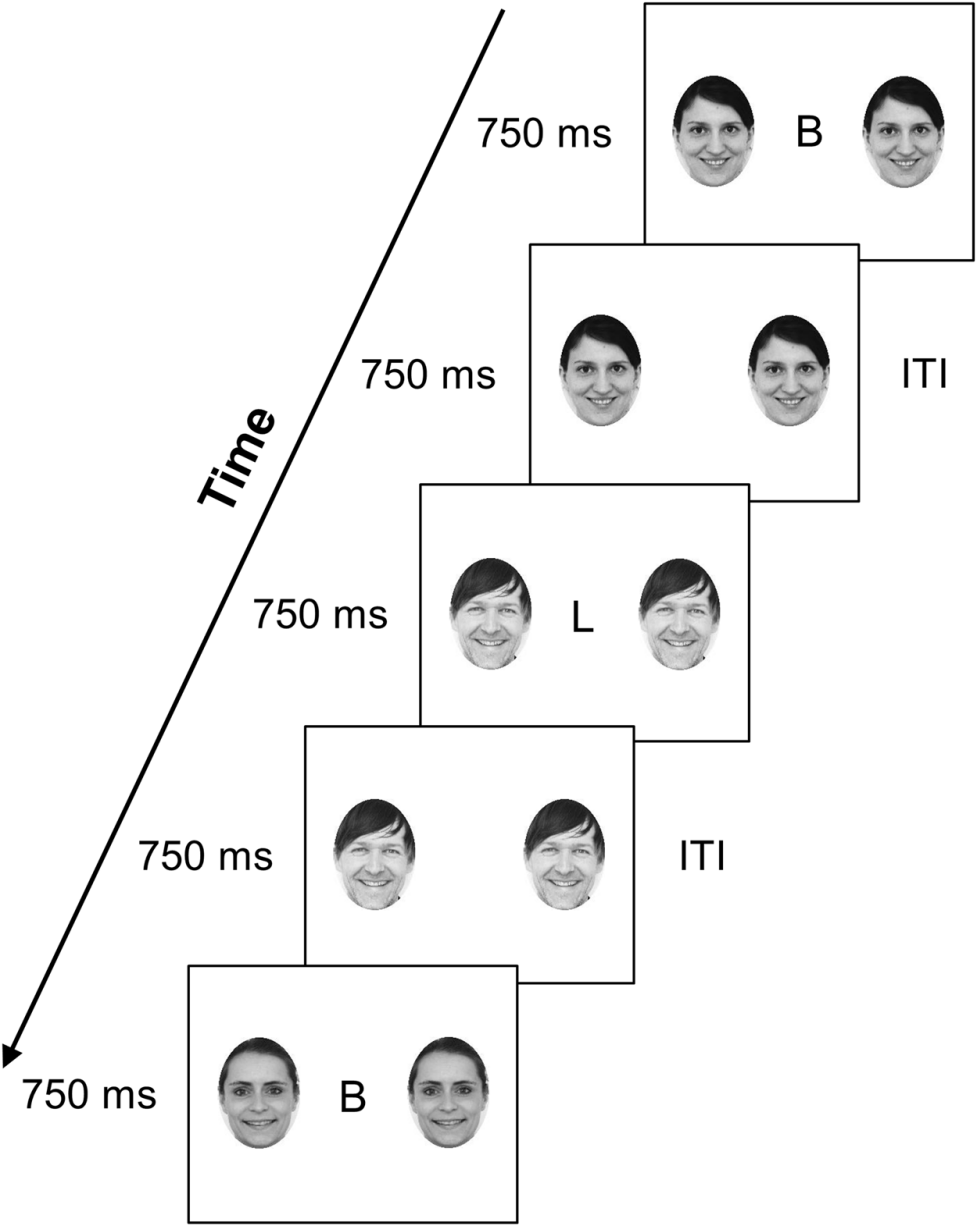
Illustration of the Emotional face n-back task. Legend: Illustrated here is an example of the happy-face 2-back condition. The participants were provided with detailed instructions regarding the 0-back and the 2-back task during a practice session. For the 0-back task, participants were instructed to press a button with their index finger when the target letter was shown. For the 2-back task, participants were instructed to press the button when the letter shown was the same as the letter shown two trials before. No reference was made to the distractors flanking the letters. Before each block, participants were informed whether the next block would be a 0-back or a 2-back condition. The face stimuli used as distractors in the experiment were color photographs of actors taken from the Karolinska Directed Emotional Faces (KDEF) set^34^ (http://www.emotionlab.se/resources/kdef). Other photographs were used in this figure for illustration purposes. ITI = intertrial interval.

During fMRI, participants performed an adapted version of the emotional face n-back task similar to that introduced by Ladouceur *et al*.^26^. In a block design, faces with different emotions (happy, angry, neutral) and Fourier-scrambled images were displayed as task-irrelevant (distractor) stimuli while participants carried out a 2-back working memory task (Fig. 1). A 0-back task with scrambled images was used as a baseline condition. The experimental paradigm included 15 blocks with 25 trials each (block duration 50 s, 15 s rest between blocks). Three blocks of each of the five conditions (2-back happy faces, 2-back angry faces, 2-back neutral faces, 2-back scrambled images, 0-back scrambled images) were presented in pseudorandomized order such that no block of the same type occurred consecutively. The face stimuli were color photographs of actors taken from the Karolinska Directed Emotional Faces (KDEF) set^34^ (additional information regarding the distractors in Supplementary Methods). Trial duration was 750 ms, the intertrial interval (ITI) also had a duration of 750 ms.

### MRI Data Acquisition and Processing

Images were acquired using standard sequences with a 3 T whole-body MRI scanner (TRIO; Siemens, Erlangen, Germany) equipped with a standard head coil. The T1-weighted structural brain scans were acquired with rapid acquisition gradient echo (MP-RAGE) sequence with the following parameters: number of slices = 176; repetition time = 1900 ms; echo time = 2.26 ms; flip angle (FA) of 9°; slice thickness of 1 mm; voxel size of 1 × 1 × 1 mm^3^; field-of view (FoV) of 256 × 224 mm^2^; bandwidth of 200 Hz/pixel. The functional images were acquired by using a gradient-echo T2*-weighted echo planar imaging (EPI) with the following parameters: tilted 30° towards AC–PC line (to reduce signal dropout in orbitofrontal regions); number of volumes = 425; number of slices = 42; repetition time = 2410 ms; echo time = 25 ms; FA of 80°; 3 mm in-plane resolution; slice thickness of 2 mm (1 mm gap resulting in a voxel size of 3 × 3 × 2 mm^3^); FoV of 192 × 192 mm^2^; bandwidth of 2112 Hz/pixel. Functional and structural images were processed using SPM8 toolbox (http://www.fil.ion.ucl.ac.uk/spm/) within the Nipype framework^35^. A DARTEL template was created using structural images from all subjects^36^. The functional images were corrected for temporal slice-timing and motion simultaneously using realign4D^37^. The six realignment parameters, describing the rigid-body movement (x, y, z, pitch, roll, yaw), were saved and later used as nuisance covariates to account for the variance due to motion. The EPI volumes were coregistered to the subject’s structural brain image. This was followed by the normalization to MNI space using the DARTEL template and corresponding flow field. The resulting data were smoothed with an isotropic 8 mm FWHM Gaussian kernel. We evaluated the quality of the fMRI data by manual inspection and using artifact detection tools (ART, https://www.nitrc.org/projects/artifact_detect/). Volumes that exceeded an intensity threshold of three standard deviations or a threshold of 2 mm normalized movement in any direction were classified as outliers. The ASD group had a mean of 2.5 (SD = 5.5) frames with motion outliers per participant, compared to a mean of 0.5 (SD = 0.8) per participant for the HC group with no significant group difference (t(22) = 1.66, p = 0.111). There were also no significant group differences regarding the number of frames with intensity outliers per participant (ASD: mean = 5.8, SD = 3.2; HC: mean = 7.5, SD = 6.1; t(35) = −1.24, p = 0.222) and the number of frames with both motion and intensity outliers per participant (ASD: mean = 0.8, SD = 2.1; HC: mean = 0.4, SD = 0.6; t(25) = 0.86, p = 0.401). After excluding motion outliers (threshold of 2 mm normalized movement), the ASD group had a mean displacement of 0.22 (SD = 0.13), compared to 0.16 (SD = 0.08) for the HC group with no significant group difference (t(41) = 1.64, p = 0.109).

### MRI Data Analysis

A General Linear Model (GLM) was fitted on the 1st level for every participant to model the hemodynamic response during five different conditions: (i) 2-back task with neutral faces, (ii) 2-back task with happy faces, (iii) 2-back task with angry faces, (iv) 2-back task with scrambled images, (v) 0-back task with scrambled images. To demonstrate the feasibility of our task and to test for potential group differences at the whole-brain level, we first contrasted (a) 2-back with scrambled images > 0-back with scrambled images and (b) 2-back with all face conditions > 2-back with scrambled images, respectively, at a threshold of p = 0.001 (uncorrected). Between-group differences had to exceed p < 0.05, family-wise error (FWE)-corrected, to guard against type I errors. To test our main hypothesis of reduced amygdala habituation in autism, we defined the bilateral amygdala as an a priori region of interest (ROI) for habituation analyses. The amygdala mask was anatomically defined by merging the left and right amygdala labels from the Automated Anatomical Labelling (AAL) atlas^38^ provided by SPM. We extracted beta values from all voxels belonging to this ROI using the MarsBar toolbox for SPM^39^. Two GLM models were fitted for habituation analyses: The first (habituation over all face conditions) was fitted on the 1st level for every participant to model the brain activation during nine conditions: nine blocks with 2-back task with faces. The second GLM (emotion-specific habituation) was fitted on the 1st level for every participant to model the brain activation during nine conditions: separately for each of the three different face conditions (2-back task with neutral faces, 2-back task with happy faces, 2-back task with angry faces) consisting of three blocks each. For all analyses, we included regressors for the 2-back task with scrambled images and the 0-back task with scrambled images, the six realignment parameters as well as motion outliers or intensity outliers as additional nuisance regressors.

### Statistical analyses

The objective of our study was to test for habituation effects in (a) behavioral data and (b) neuroimaging data, which we assessed for each patient using the regression approach first introduced to fMRI data by Plichta *et al*.^17^ and also recently applied to behavioral analysis^40^. This approach is based on the regression $Y=bX+a$. The regression coefficient *b* is an estimate of the rate of habituation and has been shown to be dependent on the intercept of the regression line *a* ^17,27^. Therefore, the absolute habituation index *b’* is a measure independent of the initial amplitude of the response. It has been determined according to Plichta *et al*.^17^ and Montagu^27^ using the formula $b\text{'}=b-c(a-\bar{a})$, in which *c* is the slope of *b* on *a* and *ā* is the mean of *a* ^17,27^. A negative value of *b’* indicates habituation.

(a) As a preliminary step, we conducted repeated measure ANOVAs on reaction time (RT) and accuracy measures (hit rate, false alarm rate) (see Supplementary Methods for definition) in order to examine the effects of socio-emotional stimuli on behavioral performance in the emotional n-back task. Stimulus type (happy faces, angry faces, neutral faces, scrambled images) and memory load (0-back and 2-back) were included as within-subjects factors and group as between-subjects factor. RT data points faster than 150 ms or slower than 1500 ms were filtered out (ASD = 0.5%, HC = 0.0%). To test for habituation effects in behavioral data, we calculated the habituation index *b’* for logarithmized RT (*b’*RT) and accuracy measures. In this analysis, Y is the block mean of logarithmized RT or accuracy measures and X is the logarithmically transformed block number of the face blocks.

(b) In our analysis of neuroimaging data, Y is the mean response in the ROI amygdala and X is the logarithmically transformed block number of the face blocks.

Group comparisons of the habituation index *b’* (for both neuroimaging and behavioral data) were performed with independent samples t-tests. All tests were performed with SPSS statistical software version 23.0 (SPSS, Chicago, Illinois), R (R Development Core Team, Vienna, Austria) or SPM 8 (http://www.fil.ion.ucl.ac.uk/spm/software/spm8).

### Data availability

The datasets generated and analyzed during the current study are available from the corresponding author on reasonable request.

## Results

### Sample characteristics

Demographic and clinical characteristics are summarized in Table 1. The groups did not differ in age or IQ. All three self-reports AQ, EQ and SQ showed significantly more autism-specific symptoms in the ASD group than in the HC group. Four patients were taking prescription medication at the time of the study (for details, see Supplementary Methods).

**Table 1:** Demographic Variables and Clinical Measures.

	N	Sample		Analyses		
	ASD/HC	ASD	HC	t	df	p
Age (Years)	22/24	34.1 ± 11.5	36.2 ± 11.0	−0.66	44	0.516
IQ	22/24	106.7 ± 14.0	113.9 ± 11.0	−1.95	44	0.058
AQ Score	22/24	35.5 ± 9.6	14.4 ± 6.4	8.72	44	<0.001*
EQ Score	22/24	18.4 ± 9.7	41.4 ± 12.4	−6.96	44	<0.001*
SQ Score	22/24	63.9 ± 20.6	47.1 ± 16.9	3.03	44	0.004*

ASD: Autism spectrum disorder; HC: Healthy controls; IQ: Intelligence quotient; AQ: Autism Spectrum Quotient; EQ: Empathy Quotient; SQ: Systemizing Quotient. Mean values ± SD for each variable are shown separately for each sample. The range for IQ was as following: ASD 85–139, HC 94–142.

### Behavioral data

Standard analyses of task performance (RT, hit rate, false alarm rate) bore out expected effects (main effect for working memory load for RT, hit rate and false alarm rate), but the groups did not differ (see Supplementary Table S2). The emotional content of the face stimuli seemed to have no impact on behavioral measures (see Supplementary Results). More importantly for the current study, habituation analyses using the habituation index *b’* yielded a significant group difference for logarithmized RT (t(44) = 2.46, p = 0.018), as HC (*b’*RT = −0.043) showed a decrement of RT over the course of the experiment but patients with ASD (*b’*RT = 0.006) did not. When including IQ as a covariate, the reported group difference remained statistically identical for *b’*RT (F(1,43) = 6.19, p = 0.017). After excluding the four ASD participants who took psychoactive medication at the time of the study, the group differences remained significant for *b’*RT (*t*(40) = 2.98, p = 0.005). There were no significant group differences for *b’* of the accuracy measures. Regarding data variability, the Bartlett test indicated homogeneity of variances for *b’*RT but unequal variances for RT (see Supplementary Results).

### Neuroimaging data

**Figure 2:**
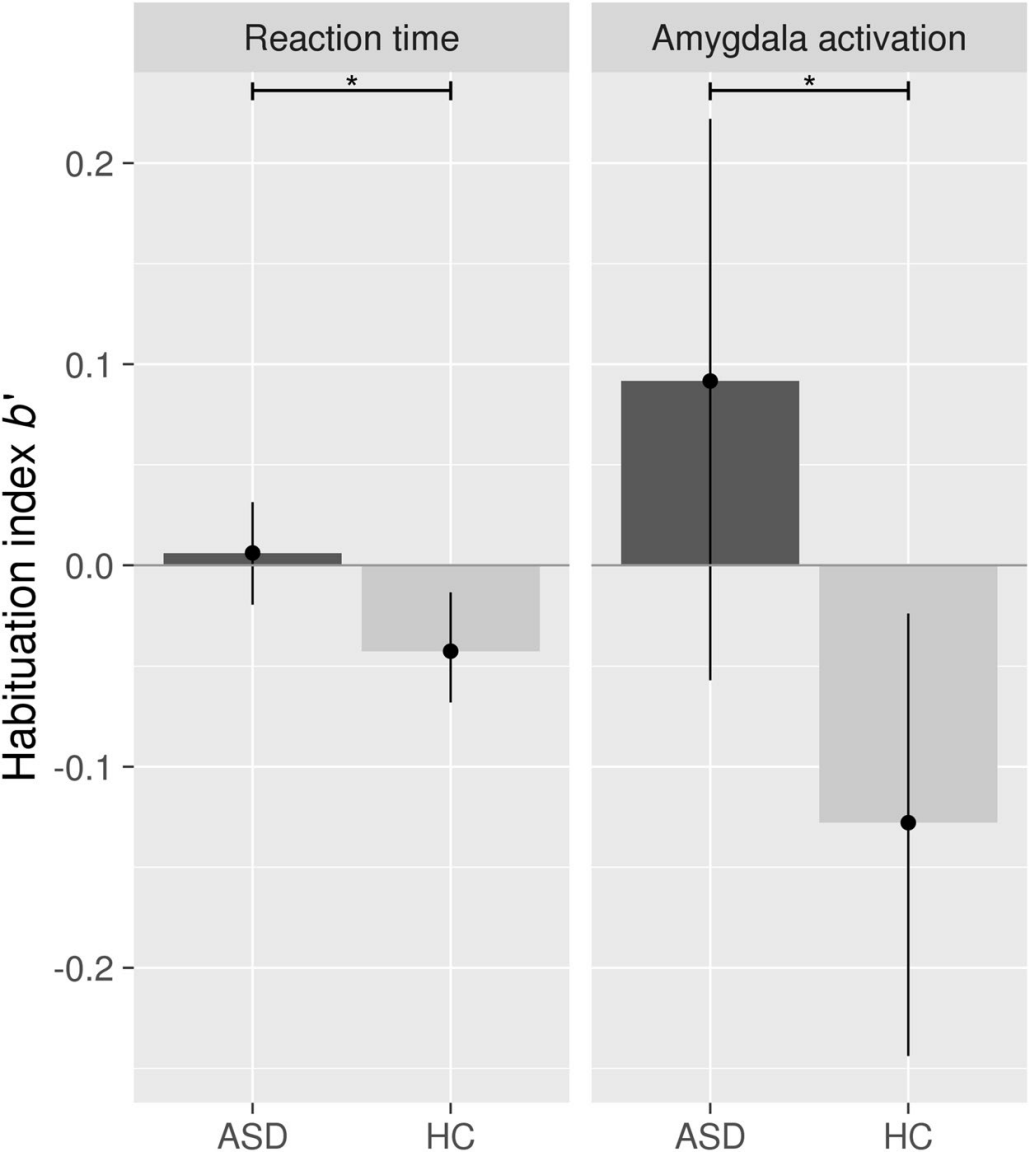
Habituation index *b’* for logarithmized reaction time (*b’*RT) and amygdala activation (*b’*amygdala). Legend: Displayed are group means and 95%-confidence intervals. A negative value of *b’* indicates habituation. Abbreviations: ASD = autism spectrum disorder, HC = healthy controls. *Independent samples t-test (p < 0.05).

Basic contrasts showed the expected main effects of working memory load and face processing in ASD as well as HC with no group differences for any of the contrasts (see Supplementary Fig. S1). Exploratory analyses of the extracted parameter estimates from the bilateral amygdala ROI did not reveal any group differences for any of the different conditions or in the face processing network (see Supplementary Results). Mirroring the lack of habituation evident in the RTs of the ASD group, amygdala habituation was evident in HC but absent in ASD. This difference was significant between groups (t(44) = 2.51, p = 0.016, Fig. 2), also when including IQ as a covariate (F(1,43) = 6.19, p = 0.017) or excluding the four ASD participants who took psychoactive medication at the time of the study (*t*(40) = 2.56, p = 0.014). Furthermore, the group difference was also unlikely to result from motion artefacts because the groups did not differ in head motion (see Methods) and follow-up analyses including additional motion parameters as covariates had no impact on the results (amygdala habituation index *b’* (*b’*amygdala): F(1,43) = 6.23, p = 0.016, see Supplementary Results for further details). To test whether this group difference was mediated by the emotional context of the face distractors, we conducted a follow-up linear mixed model on *b’*amygdala with all face conditions as within-subjects factors. The main effect of the critical difference in the between-subjects factor group remained significant (*F*(1,44) = 5.889, *p* = 0.019) and no clear effect of condition (*F*(2,88) = 2.098, *p* = 0.129) nor interaction with group emerged (*F*(2,88) = 0.157, *p* = 0.855). Regarding data variability, we found homogeneity of variances for *b’*amygdala and for amygdala activation (see Supplementary Results). Exploratory analysis of brain-behavior relationships (between the habituation indices *b’*amygdala and *b’*RT) did not reveal any significant correlations.

## Discussion

To gain insight on the potential underlying mechanisms of social impairments in ASD, we tested the notion of reduced habituation both on the level of behavioral responses as well as amygdala activation to face stimuli in a sample of adults with high-functioning ASD relative to HC subjects. To this end, we employed a 2-back working memory task flanked by faces with different emotions as task-irrelevant distractors and a habituation index *b’* recently used to test amygdala habituation^17,27^. While general working memory functioning was preserved in the current ASD sample, patients did not show a normal decrement of RT (as measured using *b’*) over the course of the experiment, suggestive of reduced behavioral habituation. Supporting this finding, we obtained evidence of altered temporal dynamics of amygdala activation in ASD, suggestive of a lack of amygdala habituation in ASD.

Regarding the finding of an abnormal decrement of RT over the course of the experiment in ASD, it is important to note that the behavioral results indicated no working memory deficit in ASD per se, as no significant group differences emerged for any of the general (non-dynamic) behavioral measures. Therefore, this finding could potentially be viewed as evidence that altered habituation in this disorder is unlikely to be a consequence of a general impairment in executive functioning. Though extensively investigated, findings on behavioral performance on working memory in ASD are heterogeneous with some studies finding impairments^41,42^ while others did not^43,44^ and seem to be influenced by the characteristics^45,46^ and complexity of the specific task. What could be reasons for altered habituation in ASD? Given that habituation tends to fail when stimuli are particularly salient or intense^20^, the lack of habituation observed in the ASD group seems to suggest that patients may have perceived the experimental stimuli, including the task-irrelevant faces, as more salient or intense relative to HC. This could be related to a detail-focused processing style in ASD as explained by the “weak central coherence theory”^47,48^. Participants with ASD may have focused on discriminating details of the stimuli instead of perceiving them as belonging to the same stimulus category, which might have led to the perception of each stimulus as novel and salient and thereby hindered behavioral habituation. Another, albeit speculative, interpretation of the observed attenuated habituation might be that it reflects a learning impairment in ASD, which can be understood in light of the “predictive coding” framework. According to this theory, perceptual experience is influenced by prior knowledge of the world shaping the individual’s predictions (priors)^49^. When reality deviates from these expectations, prediction errors occur, which triggers the shaping of future, more optimal priors^50^. Individuals with ASD might have either attenuated^49^ or very strong^50^ priors, leading to more unexpected variability^49^. Applied to the current behavioral data, HC might have acquired the prior that faces will be of no relevance to the experimental task. Thus, they may have become increasingly effective in filtering out task-irrelevant details (which can be viewed as a form of learning) and using their cognitive resources to enhance speed in the working memory task. In contrast, the ASD group might have been unable to form priors of adequate precision.

One possible interpretation for the observed lack of amygdala habituation in the ASD group is that it may be reflective of difficulties in the extraction and interpretation of social information, and in differentiating between salient and non-salient information^25^. From a developmental perspective, impaired amygdala habituation may contribute to avoidance of faces and social interactions and thereby to atypicalities in brain networks involved in social cognition in ASD^25^. Alternatively, altered amygdala habituation might have been influenced by anxiety due to the unfamiliarity and the limited predictability of the test situation. Acute stress has been shown to trigger increased responsiveness of the amygdala^51^. Furthermore, in ASD, anxiety symptoms have been linked to attentional capture by peripherally presented faces^52^. It has also been suggested that an impairment of predictive abilities (see also above) in ASD makes the world appear overwhelming and could account for a number of typical features of ASD, including insistence on sameness, sensory hypersensitivities and difficulties with theory of mind^53^. This hypothesis is supported by evidence of impaired habituation in other modalities, as the key determinant of habituation is stimulus predictability^53^. For instance, Guiraud *et al*.^54^ found that a group of infants at high risk for autism showed reduced neural habituation to repeated sounds compared to a control group. While the underlying mechanisms of such a prediction impairment are still unclear, the predictive coding framework, which suggests a lack of priors of adequate precision as driving factor, could be a possible, albeit speculative explanation for the lack of amygdala habituation observed in our participants with ASD. Yet another conceivable explanation may be differences regarding Hebbian (associative) learning between the ASD and the HC groups. Hebbian learning algorithms explain learning in neural networks (“units that fire together, wire together”) and appear to occur incidentally and without any explicit task or attempt to learn^55^.

Our finding of a lack of amygdala habituation in ASD is generally in line with previous findings that investigated habituation by comparing mean activation in the second versus the first half of an experiment. The most recent study on this topic by Kleinhans *et al*.^25^ showed increased amygdala activation to fearful faces in run 2 versus run 1 in the ASD group while the HC group showed the expected decrease in activation over time. In an earlier study, Kleinhans *et al*. found amygdala habituation to neutral faces in HC but only a marginal decrease in amygdala activation in subjects with ASD^3^. As a possible explanation for this difference between their two studies, Kleinhans *et al*. stated that the shorter stimulus presentation time (23 ms) in their more recent study might have led ASD participants to not engage the amygdala during the first run due to slower, inefficient information processing in ASD^25^. In our study, every face stimulus was displayed for a total of 1.5 s during the trial and subsequent ITI, which should have provided all participants with sufficient processing time. This is supported by exploratory analyses which did not reveal any group differences of neural activation in the face processing network. As habituation can be defined as decrement of response to irrelevant and inconsequential stimuli, displaying faces as task-relevant and therefore important stimuli might possibly affect habituation. We addressed this problem by choosing a 2-back working memory task and displaying faces as task-irrelevant distractors. To the best of our knowledge, there has only been one other study to date with a somewhat similar approach that has instructed participants to react to a fixation cross rather than to the images of faces displayed during the experimental task^25^.

Our study included face stimuli with happy, angry and neutral expressions as distractors. We found no significant interaction between group and condition, supporting our hypothesis that temporal dynamics of amygdala activation are robust for both emotional and neutral face stimuli. While the amygdala responds to all visual emotional stimuli, it shows a stronger activation for faces, which demonstrates its role in face perception^6,7^. There is strong evidence for the amygdala being a part of a cortical network for face perception with a crucial role in processing facial expressions^56^ and salient social information^57^. In line with the current finding, Kleinhans *et al*. found evidence that measures of atypical neural dynamics in ASD may be especially robust for both neutral and emotional face stimuli^25^. Swartz *et al*. included faces expressing different emotions (sad, fearful, happy, neutral) in their study^24^. Only partly consistent with our results, they found that reduced habituation in youth with ASD was specific to sad and neutral faces while both the ASD and the HC group did not habituate to fearful or happy faces^24^. The results of our study indicate that when face stimuli are displayed as task-irrelevant distractors, their emotional content seems to have a negligible effect on temporal dynamics of amygdala activation. However, it is important to note that our data do not unequivocally demonstrate that amygdala habituation in ASD is specific for socially relevant stimuli (such as the face images used here). Future targeted studies might need to modify the fMRI paradigm to also include other stimulus categories. Furthermore, the relationship between behavioral and neural habituation remains unclear.

To the best of our knowledge, our study was the first to employ a measure of habituation using a regression approach and adjusting for initial activation amplitude^17,27^ to test amygdala habituation in ASD. As Plichta *et al*.^17^ argued, comparing means of amygdala activation amplitude might be an oversimplification, and thus might not accurately describe habituation. Assessing amygdala habituation during a facial emotion matching paradigm in a group of healthy subjects, Plichta *et al*.^17^ found higher within-subject reliability for the habituation index than for standard analyses of mean response amplitude. Analogous to a recent study that applied the *b’* index to describe the temporal dynamics of a behavioral measure, we used *b’* to test for habituation effects in RT.

When considering our findings, some important limitations have to be taken into account. First, the design of our experiment with a pseudorandomized order of blocks of face stimuli with different emotions that varies between patients may pose a limitation for emotion-specific analyses of habituation. However, the current study was motivated largely by the study of Plichta *et al*.^17^ which also conducted epoch-related analyses. Furthermore, other influential studies of amygdala habituation in ASD also employed block designs^3,24,25^. Future studies might benefit from event-related designs as they might provide a finer-grained picture of habituation and related phenomena such as repetition suppression^58^. Moreover, our experimental design cannot definitively demonstrate that the observed lack of amygdala habituation in ASD was specific to faces. The design was also not suitable to verify or reject the hypothesis regarding the predictive coding framework. Second, the recruitment of the participants with ASD through the autism outpatient clinic and local self-help groups may have led to a selection bias. Furthermore, our sample was somewhat older than other studies in adults with ASD. Third, a factor to consider when interpreting our results is that we did not collect eye-tracking data. It is possible that gaze patterns were also influenced by the fact that the face stimuli were task-irrelevant. However, since neural activation was present in face-sensitive brain areas and we found no group difference in these areas, we consider the possibility of such a bias to be of minor significance. Fourth, head motion can be a possible confounding factor in fMRI studies but is unlikely to have had a significant impact on the main findings as demonstrated by group comparisons of various motion parameters and further analyses using additional motion parameters as covariates. Critically, in contrast to previous studies^3,24,25^, we found no relationship between the habituation index and clinical measures including AQ score. Future research is therefore needed to understand relationships with symptoms and functioning.

In conclusion, the current study provides further evidence for altered temporal dynamics on the level of behavioral responses and amygdala activity in ASD. Developing a broader comprehension of the extent of habituation impairments as well as the underlying emotional and perceptual mechanisms is needed to understand abnormalities in perception and social impairment in ASD.

## Electronic supplementary material

Supplementary Information

## References

[1] American Psychiatric Association. *Diagnostic And Statistical Manual Of Mental Disorders* (*5th ed*.). (American Psychiatric Publishing, Arlington, USA, 2013).

[2] Kennedy DP, Adolphs R (2012). Perception of emotions from facial expressions in high-functioning adults with autism. Neuropsychologia.

[3] Kleinhans NM (2009). Reduced neural habituation in the amygdala and social impairments in autism spectrum disorders. Am. J. Psychiat..

[4] Baron-Cohen S (2000). The amygdala theory of autism. Neurosci. Biobehav. Rev..

[5] Phelps EA, LeDoux JE (2005). Contributions of the amygdala to emotion processing: from animal models to human behavior. Neuron.

[6] Sergerie K, Chochol C, Armony JL (2008). The role of the amygdala in emotional processing: a quantitative meta-analysis of functional neuroimaging studies. Neurosci. Biobehav. Rev..

[7] Hariri AR, Tessitore A, Mattay VS, Fera F, Weinberger DR (2002). The amygdala response to emotional stimuli: a comparison of faces and scenes. Neuroimage.

[8] Haxby JV, Hoffman EA, Gobbini MI (2000). The distributed human neural system for face perception. Trends Cogn. Sci..

[9] Schultz RT (2005). Developmental deficits in social perception in autism: the role of the amygdala and fusiform face area. Int. J. Dev. Neurosci..

[10] Wiggins JL, Swartz JR, Martin DM, Lord C, Monk CS (2014). Serotonin transporter genotype impacts amygdala habituation in youth with autism spectrum disorders. Soc. Cogn. Affect. Neurosci..

[11] Baron-Cohen S (1999). Social intelligence in the normal and autistic brain: an fMRI study. Eur. J. Neurosci..

[12] Hadjikhani N, Joseph RM, Snyder J, Tager-Flusberg H (2007). Abnormal activation of the social brain during face perception in autism. Hum. Brain Mapp..

[13] Monk CS (2010). Neural circuitry of emotional face processing in autism spectrum disorders. J. Psychiatry Neurosci..

[14] Weng S-J (2011). Neural activation to emotional faces in adolescents with autism spectrum disorders. J. Child Psychol. Psychiatry.

[15] Pierce K, Haist F, Sedaghat F, Courchesne E (2004). The brain response to personally familiar faces in autism: findings of fusiform activity and beyond. Brain.

[16] Pierce K, Müller R-A, Ambrose J, Allen G, Courchesne E (2001). Face processing occurs outside the fusiform ‘face area’ in autism: evidence from functional MRI. Brain.

[17] Plichta MM (2014). Amygdala habituation: a reliable fMRI phenotype. Neuroimage.

[18] Phillips ML (2001). Time courses of left and right amygdalar responses to fearful facial expressions. Hum. Brain Mapp..

[19] Thompson RF, Spencer WA (1966). Habituation: a model phenomenon for the study of neuronal substrates of behavior. Psychol. Rev..

[20] Rankin CH (2009). Habituation revisited: an updated and revised description of the behavioral characteristics of habituation. Neurobiol. Learn. Mem..

[21] Ramaswami M (2014). Network plasticity in adaptive filtering and behavioral habituation. Neuron.

[22] Breiter HC (1996). Response and habituation of the human amygdala during visual processing of facial expression. Neuron.

[23] Wedig MM, Rauch SL, Albert MS, Wright CI (2005). Differential amygdala habituation to neutral faces in young and elderly adults. Neurosci. Lett..

[24] Swartz JR, Wiggins JL, Carrasco M, Lord C, Monk CS (2013). Amygdala habituation and prefrontal functional connectivity in youth with autism spectrum disorders. J. Am. Acad. Child Adolesc. Psychiatry.

[25] Kleinhans NM, Richards T, Greenson J, Dawson G, Aylward E (2016). Altered dynamics of the fMRI response to faces in individuals with autism. J. Autism Dev. Disord..

[26] Ladouceur CD (2009). Fearful faces influence attentional control processes in anxious youth and adults. Emotion.

[27] Montagu JD (1963). Habituation of the psycho-galvanic reflex during serial tests. J. Psychosom. Res..

[28] Harris PA (2009). Research electronic data capture (REDCap)–a metadata-driven methodology and workflow process for providing translational research informatics support. J. Biomed. Inform..

[29] Baron-Cohen S, Wheelwright S, Skinner R, Martin J, Clubley E (2001). The autism-spectrum quotient (AQ): evidence from Asperger syndrome/high-functioning autism, males and females, scientists and mathematicians. J. Autism Dev. Disord..

[30] Baron-Cohen S, Wheelwright S (2004). The empathy quotient: an investigation of adults with Asperger syndrome or high functioning autism, and normal sex differences. J. Autism Dev. Disord..

[31] Baron-Cohen S, Richler J, Bisarya D, Gurunathan N, Wheelwright S (2003). The systemizing quotient: an investigation of adults with Asperger syndrome or high-functioning autism, and normal sex differences. Philos. Trans. R. Soc. Lond. B. Biol. Sci..

[32] von Aster, M., Neubauer, A. & Horn, R. *WIE - Wechsler Intelligenztest Für Erwachsene*. (Huber, Bern, Switzerland, 2006).

[33] Donnell AJ, Pliskin N, Holdnack J, Axelrod B, Randolph C (2007). Rapidly-administered short forms of the Wechsler Adult Intelligence Scale-3rd edition. Arch. Clin. Neuropsychol..

[34] Lundqvist, D., Flykt, A. & Öhman, A. *The Karolinska directed emotional faces* (*KDEF*). (CD ROM from Department of Clinical Neuroscience, Psychology section, Karolinska Institutet, 1998).

[35] Gorgolewski, K. *et al*. Nipype: a flexible, lightweight and extensible neuroimaging data processing framework in Python. *Front*. *Neuroinformatics* **5**, 10.3389/fninf.2011.00013 (2011). 10.3389/fninf.2011.00013 PMC3159964 21897815

[36] Ashburner J (2007). A fast diffeomorphic image registration algorithm. Neuroimage.

[37] Roche A (2011). A four-dimensional registration algorithm with application to joint correction of motion and slice timing in fMRI. IEEE Trans. Med. Imaging.

[38] Tzourio-Mazoyer N (2002). Automated anatomical labeling of activations in SPM using a macroscopic anatomical parcellation of the MNI MRI single-subject brain. Neuroimage.

[39] Brett M, Anton J, Valabregue R, Poline J (2002). Region of interest analysis using the MarsBar toolbox for SPM 99. Neuroimage.

[40] Avery SN, VanDerKlok RM, Heckers S, Blackford JU (2016). Impaired face recognition is associated with social inhibition. Psychiatry Res..

[41] Chen S-F (2016). Deficits in executive functions among youths with autism spectrum disorders: an age-stratified analysis. Psychol. Med..

[42] Fried R (2016). A study of the neuropsychological correlates in adults with high functioning autism spectrum disorders. Acta Neuropsychiatr..

[43] Koshino H (2005). Functional connectivity in an fMRI working memory task in high-functioning autism. Neuroimage.

[44] Ozonoff S, Strayer DL (2001). Further evidence of intact working memory in autism. J. Autism Dev. Disord..

[45] Nakahachi T (2006). Discrepancy of performance among working memory-related tasks in autism spectrum disorders was caused by task characteristics, apart from working memory, which could interfere with task execution. Psychiatry Clin. Neurosci..

[46] Williams DL, Goldstein G, Carpenter PA, Minshew NJ (2005). Verbal and spatial working memory in autism. J. Autism Dev. Disord..

[47] Happé F, Frith U (2006). The weak coherence account: detail-focused cognitive style in autism spectrum disorders. J. Autism Dev. Disord..

[48] Chan JS, Naumer MJ (2014). Explaining autism spectrum disorders: central coherence vs. predictive coding theories. J. Neurophysiol..

[49] Pellicano E, Burr D (2012). When the world becomes ‘too real’: a Bayesian explanation of autistic perception. Trends Cogn. Sci..

[50] Cruys SV, de, de-Wit L, Evers K, Boets B, Wagemans J (2013). Weak priors versus overfitting of predictions in autism: reply to Pellicano and Burr (TICS, 2012). Iperception.

[51] van Marle HJF, Hermans EJ, Qin S, Fernández G (2009). From specificity to sensitivity: how acute stress affects amygdala processing of biologically salient stimuli. Biol. Psychiatry.

[52] Herrington, J. D. *et al*. Negative valence in autism spectrum disorder: the relationship between amygdala activity, selective attention, and co-occurring anxiety. *Biol*. *Psychiatry Cogn*. *Neurosci*. *Neuroimaging*, 10.1016/j.bpsc.2017.03.009 (2017). 10.1016/j.bpsc.2017.03.009 PMC6124303 29348040

[53] Sinha P (2014). Autism as a disorder of prediction. Proc. Natl. Acad. Sci. USA.

[54] Guiraud JA (2011). Differential habituation to repeated sounds in infants at high risk for autism. Neuroreport.

[55] Munakata Y, Pfaffly J (2004). Hebbian learning and development. Dev. Sci..

[56] Ishai A (2008). Let’s face it: it’s a cortical network. Neuroimage.

[57] Skelly, L. R. & Decety, J. Passive and motivated perception of emotional faces: qualitative and quantitative changes in the face processing network. *PLOS ONE* **7**, 10.1371/journal.pone.0040371 (2012). 10.1371/journal.pone.0040371 PMC3386961 22768287

[58] Nordt M, Hoehl S, Weigelt S (2016). The use of repetition suppression paradigms in developmental cognitive neuroscience. Cortex.

