# Supplementary material for: Altered behavioral and amygdala habituation in high-functioning adults with autism spectrum disorder: an fMRI study
Source: Sci Rep. 2017 Oct 19;7:13611. doi: 10.1038/s41598-017-14097-2 (PMC5648793; doi:10.1038/s41598-017-14097-2)
Supplement: Supplementary file 1 — Supplementary Information [file 41598_2017_14097_MOESM1_ESM.pdf]

Supplementary Information for Altered behavioral and amygdala habituation in high-functioning adults with autism spectrum disorder: an fMRI study

Authors:

Friederike I. Tam[1,3] (friederike.tam@uniklinikum-dresden.de)

Joseph A. King[1] (joseph.king@uniklinikum-dresden.de)

Daniel Geisler[1] (daniel.geisler@uniklinikum-dresden.de)

Franziska M. Korb[4] (franziska.korb@tu-dresden.de)

Juliane Sareng[1,3] (juliane.sareng@uniklinikum-dresden.de)

Franziska Ritschel[1,2] (franziska.ritschel@uniklinikum-dresden.de)

Julius Steding[1] (julius.steding@uniklinikum-dresden.de)

Katja U. Albertowski[3] (katja.albertowski@uniklinikum-dresden.de)

Veit Roessner[3] (veit.roessner@uniklinikum-dresden.de)

*Stefan Ehrlich[1,2] (stefan.ehrlich@uniklinikum-dresden.de), Corresponding author

[1] Division of Psychological and Social Medicine and Developmental Neurosciences, Faculty of Medicine, Technische Universität Dresden, Fetscherstraße 74, 01307 Dresden, Germany

[2] Translational Developmental Neuroscience Section, Eating Disorder Research and Treatment Center at the Department of Child and Adolescent Psychiatry, Faculty of Medicine, Technische Universität Dresden, Fetscherstraße 74, 01307 Dresden, Germany

[3] Department of Child and Adolescent Psychiatry, Faculty of Medicine, University Hospital C. G. Carus, Technische Universität Dresden, Fetscherstraße 74, 01307 Dresden, Germany

[4] Department of Psychology, Technische Universität Dresden, Zellescher Weg 17, 01069 Dresden, Germany

Supplementary Methods

Participants

Participants of both groups were excluded if they had a history of any of the following diagnoses: organic brain syndrome, epilepsy, dementia, schizophrenia, psychosis NOS, bipolar disorder or substance dependence. Further exclusion criteria for all participants were intelligence quotient (IQ) <85, current substance abuse (including alcohol), current moderate to severe depression, current acute inflammatory, neurologic or metabolic illness, pregnancy or breast feeding. Healthy controls (HC) were excluded if they had any history of psychiatric illness. Regarding psychiatric comorbidities in the autism spectrum disorder (ASD) group, 10/22 participants reported symptoms of depression in their lifetime (one patient was treated with sertraline and one with agomelatine at the time of the study), 1/22 reported symptoms of anxiety and 3/22 had been diagnosed with attention deficit hyperactivity disorder or attention deficit disorder (two patients were treated with methylphenidate at the time of the study). None of the participants had been diagnosed with obsessive-compulsive disorder. Supplementary Table S1 notes all prescription medication used at the time of the study.

Supplementary Table S1. Prescription medication at the time of the study.

Participant	Group	Medication (active ingredient if known)	Indication
1	ASD	*Methylphenidate	Attention deficit (hyperactivity) disorder
2	ASD	*Methylphenidate<br>Chlorphenamine	Attention deficit (hyperactivity) disorder<br>Allergic symptoms
3	ASD	*Agomelatine	Depressive symptoms
4	ASD	*Sertraline	Depressive symptoms
5	ASD	Ramipril	Hypertension
6	ASD	Metformin<br>ACE inhibitor	Type 2 diabetes mellitus<br>Hypertension
7	HC	Antibiotic	Respiratory infection (last dose)
8	HC	Levothyroxine	Hypothyroidism
9	HC	Ramipril	Hypertension
10	HC	Ethinyl estradiol/ Levonorgestrel	Oral contraceptive

ASD: Autism spectrum disorder; HC: Healthy controls. Psychoactive medications are marked with* in the column “Medication”.

Experimental paradigm: presentation of distractors

Each participant completed three blocks of each of the five conditions (2-back happy faces, 2-back angry faces, 2-back neutral faces, 2-back scrambled images, 0-back scrambled images), with a total of 75 stimuli per condition. A total of 200 different face stimuli were used (67 happy faces, 65 angry faces and 68 neutral faces). Each identity produced a happy face, an angry face and a neutral face; i.e. there were a total 65-68 different identities. For each condition and participant, stimuli were shuffled in the beginning of the experiment and then presented consecutively until no stimuli of this condition were left. Then, the stimuli were shuffled and displayed according to the same algorithm again, so that only a fraction of individual stimuli were presented twice over the course of the experiment.

Behavioral measures

For all behavioral measures, the first trial in a block was excluded and only valid button presses, defined by a reaction time (RT) between 150ms and 1500ms after stimulus presentation, were included. The hit rate was defined as the number of hits divided by the number of targets. The false-alarm rate was defined as the number of false alarms divided by the number of non-targets.

Supplementary Results

Behavioral data

To investigate if the emotional content of the face stimuli had an impact on behavioral measures, we conducted an ANOVA including all face conditions (happy faces, angry faces, neutral faces) as within-subjects factors and group as between-subjects factor. For RT, we found no significant main effect for condition and no significant interaction between group and condition but a trend for group ($F(1,1)=3.762$, $p=0.059$) with participants of the ASD group having longer RTs than the HC group. There were no significant effects for hit rate or false alarm rate.

Supplementary Table S2. Behavioral data.

Behavioral measure	Sample	
task type, distractor type	ASD	HC
Reaction time		
0-back task, scrambled images	476.2 ± 80.0	456.2 ± 67.5
2-back task, scrambled images	502.1 ± 113.8	476.5 ± 71.2
2-back task, all faces	519.7 ± 110.0	468.5 ± 67.2
2-back task, emotional faces	521.1 ±111.4	471.1 ± 69.1
2-back task, neutral faces	518.0 ±118.3	462.3 ± 70.4
Hit rate		
0-back task, scrambled images	0.99 ± 0.03	1.00 ± 0.02
2-back task, scrambled images	0.92 ± 0.09	0.94 ± 0.09
2-back task, all faces	0.93 ± 0.07	0.93 ± 0.05
2-back task, emotional faces	0.93 ± 0.07	0.93 ± 0.06
2-back task, neutral faces	0.93 ± 0.10	0.94 ± 0.05
False alarm rate		
0-back task, scrambled images	0.004 ± 0.010	0.002 ± 0.006
2-back task, scrambled images	0.030 ± 0.035	0.015 ± 0.019
2-back task, all faces	0.028 ± 0.034	0.015 ± 0.017
2-back task, emotional faces	0.030 ± 0.037	0.014 ± 0.017
2-back task, neutral faces	0.024 ± 0.033	0.017 ± 0.021

Abbreviations: ASD: autism spectrum disorder; HC: healthy controls. Mean values ± SD for each variable are shown separately for each sample. Neither the presentation of faces in comparison to scrambled images, nor the comparison between faces with emotional and faces with neutral expressions had a significant effect on reaction time, hit rate or false alarm rate.

Neuroimaging data

Exploratory whole-brain analyses (one-sample t-tests) of working memory load (contrast 2-back with scrambled images > 0-back with scrambled images) and face processing (contrast 2-back with all face conditions > 2-back with scrambled images) confirmed that our task elicited expected activation patterns in commonly associated brain regions (Supplementary Figure S1). However, no group differences (two-sample t-tests) were evident anywhere in the brain at a family-wise error (FWE)-corrected threshold of $p<0.05$. Additionally, we extracted parameter estimates from the bilateral amygdala region of interest (ROI) for the five different conditions included in our basic General Linear Model (GLM) (2-back task with neutral faces, 2-back task with happy faces, 2-back task with angry faces, 2-back task with scrambled images, 0-back task with scrambled images). Two-sample t-tests did not reveal group differences for any condition (2-back task with neutral faces, 2-back task with happy faces, 2-back task with angry faces, 2-back task with scrambled images), for the working memory load contrast (2-back with scrambled images > 0-back with scrambled images), for the face processing contrast (2-back with all face conditions > 2-back with scrambled images) or the contrast exploring the emotional load of facial expressions (2-back with emotional face conditions, i. e. angry, happy > 2-back with neutral faces). The level of significance was set to $p=0.05$ (FWE-corrected).

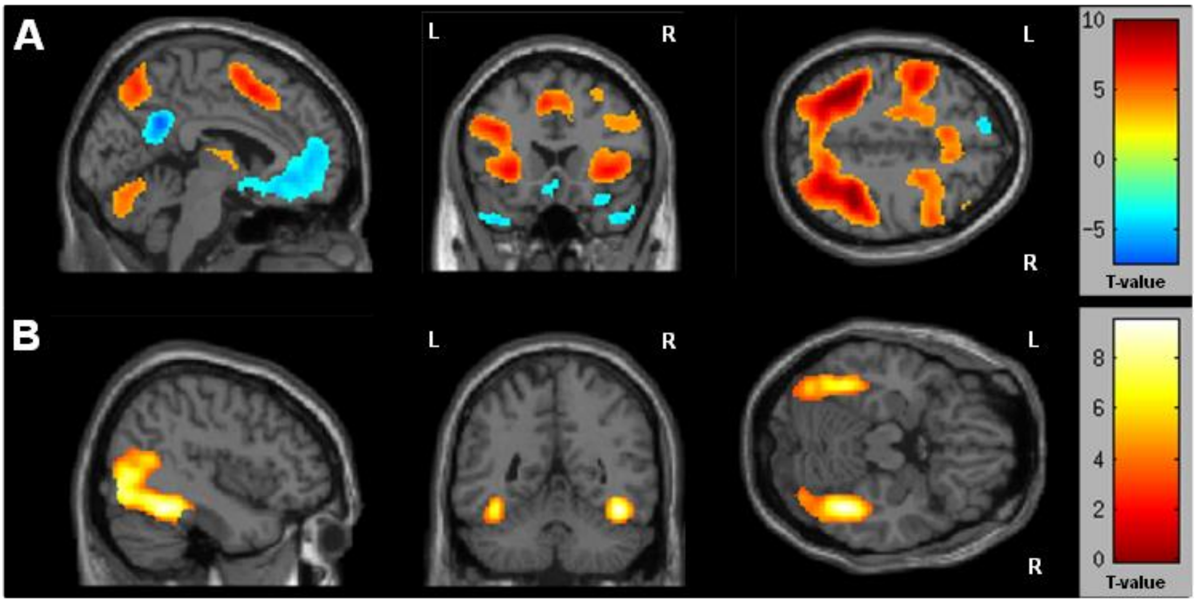

Supplementary Figure S1. Results of exploratory whole-brain analyses designed to confirm that the employed task elicited expected activation patterns associated with working memory load (Panel A) and face processing (Panel B). Panel A: Activation map for the contrast 2-back with scrambled images > 0-back (p=0.001, cluster size=50, uncorrected) over all participants with activation in the typical working memory-related brain regions DLPFC, VLPFC and mediofrontal wall[1], no group differences between ASD group and HC group emerged (correction with FWE at p=0.05). Slices are located at the following MNI coordinates (-4,20,42). Panel B: Activation map for the contrast 2-back with all face conditions > 2-back with scrambled images (p=0.001, uncorrected) over all participants with bilateral activation in face-sensitive areas[2], no group differences between ASD group and HC group emerged (correction with FWE at p=0.05). Slices are located at the following MNI coordinates (44,-50,-18). Results were visualized using xjView toolbox (http://www.alivelearn.net/xjview). Supplementary Table S3 provides more details.

Supplementary Table S3. Whole-brain results.

	Cluster			Peak			
	p (FWE-corr)	k	p (unc)	X	Y	Z	T
**Working memory load (Panel A)**							
Parietal (including Brodmann 40+7)	<0.001	11048	<0.001	40	-46	46	9.90
				-36	-52	44	9.84
				-40	-42	44	9.67
Inferior/middle frontal	<0.001	18454	<0.001	-42	2	36	9.48
				-46	28	28	8.75
				-24	0	52	8.42
Cerebellum	<0.001	3142	<0.001	28	-62	-24	8.74
				-30	-62	-26	7.62
				10	-74	-22	6.46
**Face processing (Panel B)**							
Temporal, Inferior/middle occipital (including Brodmann 19+37), Fusiform	<0.001	2566	<0.001	-44	-78	2	9.50
				-40	-54	-14	7.83
				-38	-70	-12	7.82
Temporal, Inferior/middle occipital (including Brodmann 19+37), Fusiform	<0.001	3671	<0.001	42	-52	-18	9.45
				46	-72	2	8.34
				42	-74	-10	7.97

Whole brain results for the contrasts working memory load (2-back with scrambled images > 0-back with scrambled images) and face processing (2-back with all face conditions > 2-back with scrambled images) at a threshold of p<0.05 family-wise error (FWE)-corrected, coordinates in Montreal Neurological Institute (MNI) space [x,y,z]. Clusters with a k>50 are included in the table. Abbreviations: FWE-corr: family-wise error corrected; unc: uncorrected.

Additional analyses

Differences in variability are unlikely to drive the observed group differences for habituation measures: Welch two-sample t-tests yielded no differences between the ASD and the HC groups for the coefficients of variation (CV) of RT (ASD: CV=0.13, HC: CV=0.13, $t(43)=0.49$, p=0.627) and amygdala activation (ASD: CV=1.63, HC: CV=0.48, $t(44)=0.46$, p=0.646). Additionally, tests of equality of variances were conducted for RT, amygdala activation, *b'*RT (*b'* for reaction time) and *b'*amygdala (*b'* for amygdala activation) (Bartlett test for normal distribution, Levene test for non-normal distribution). For RT (Shapiro-Wilks normality test: ASD: p=0.149, HC: p=0.649), the Bartlett test indicated unequal variances (K^2=5.28, df=1, p=0.021). For amygdala activation (Shapiro-Wilks normality test: ASD: p=0.382, HC: p=0.040), the

Levene test showed homogeneity of variances (F(1,44)=0.09, p=0.760). For *b'*RT (Shapiro-Wilks normality test: ASD: p=0.889, HC: p=0.953), the Bartlett test showed homogeneity of variances (K^2=0.54, df=1, p=0.462). For *b'*amygdala (Shapiro-Wilks normality test: ASD: p=0.002, HC: p=0.294), the Levene test showed homogeneity of variances (F(1,44)<0.01, p=0.999). Supplementary Figures S2 to S5 show box plots per group for these measures.

Furthermore, head motion was investigated as a possible confounding factor for the group difference for the habituation measures *b'*RT and *b'*amygdala. Both mean rotation (ASD: mean=0.006°, SD=0.004; HC: mean=0.005°, SD=0.002; t(33)=0.68, p=0.499) and mean translation (ASD: mean=0.381mm, SD=0.350; HC: mean=0.326mm, SD=0.187; t(32)=0.66, p=0.516) did not differ significantly between groups. When including mean displacement as a covariate, the reported group differences for *b'*RT and *b'*amygdala remained statistically significant (*b'*RT: F(1,43)=5.90, p=0.019; *b'*amygdala: F(1,43)=6.23, p=0.016). Similarly, including the other motion measures (number of motion outlier artifacts per subject, mean rotation and mean translation) as covariates had no impact on the group differences for *b'*RT and *b'*amygdala.

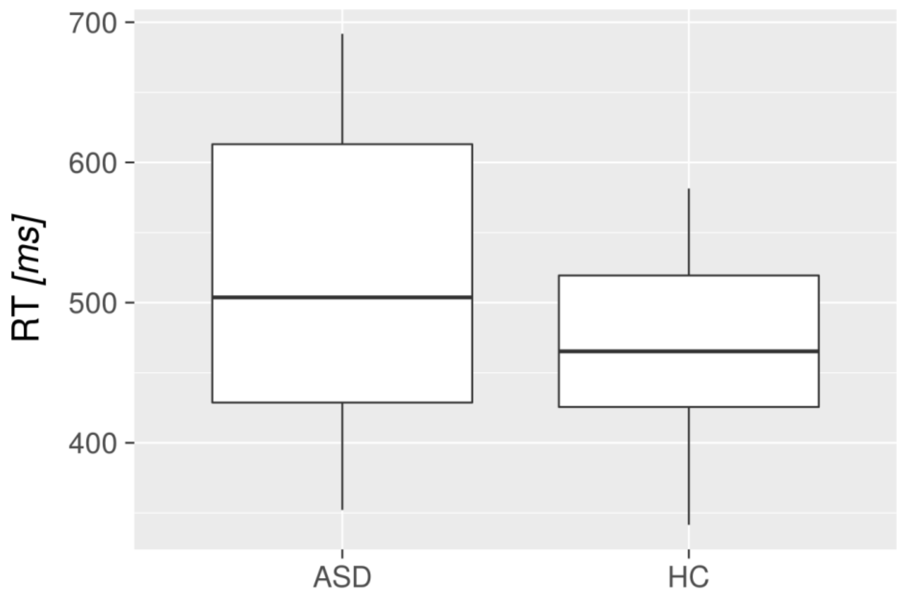

Supplementary Figure S2: Box plot for reaction time, showing the median, first and third quartile. Abbreviations: RT=reaction time, ASD=autism spectrum disorder, HC=healthy controls.

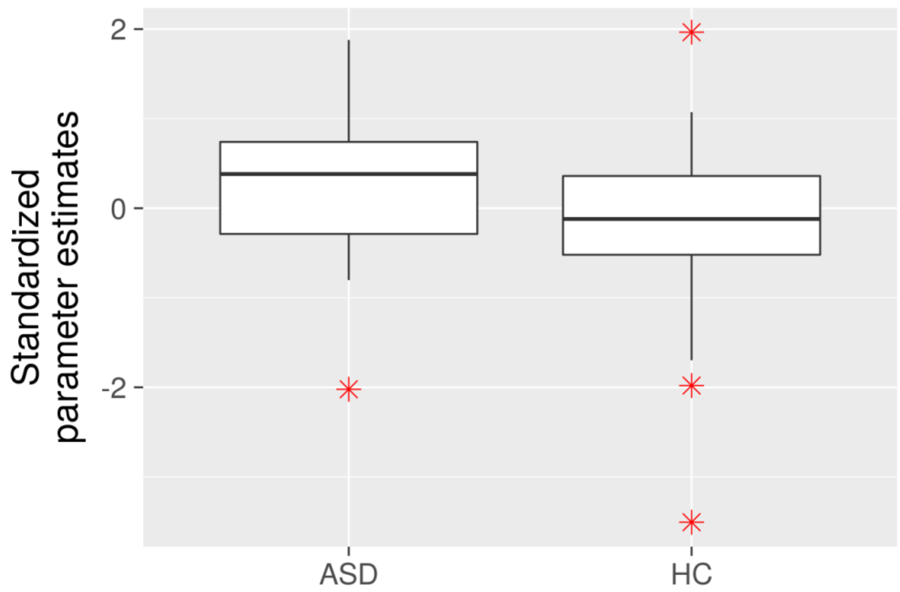

Supplementary Figure S3: Box plot for amygdala activation (z-standardized parameter estimates), showing the median, first and third quartile and outliers (asterisks, defined as 1.5 interquartile ranges below the first quartile or above the third quartile). Abbreviations: ASD=autism spectrum disorder, HC=healthy controls.

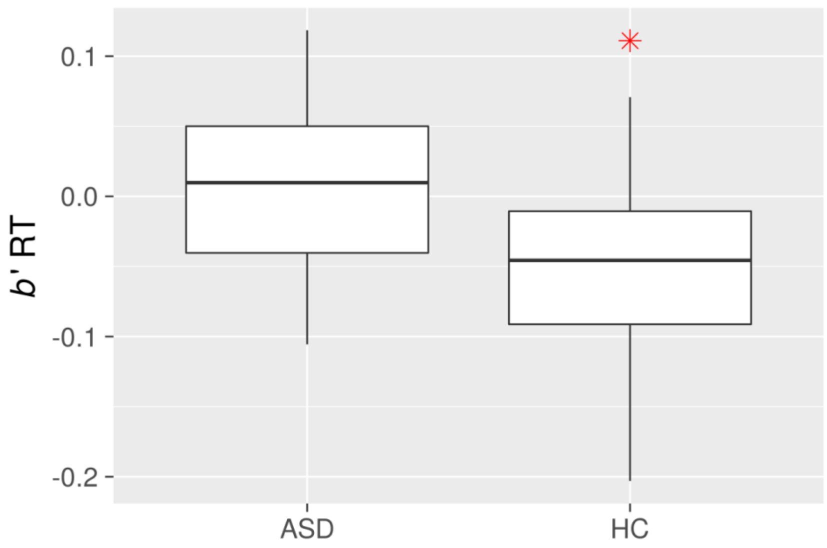

Supplementary Figure S4: Box plot for *b'* for reaction time, showing the median, first and third quartile and outliers (asterisks, defined as 1.5 interquartile ranges below the first quartile or above the third quartile). Abbreviations: *b'*RT= *b'* for reaction time, ASD=autism spectrum disorder, HC=healthy controls.

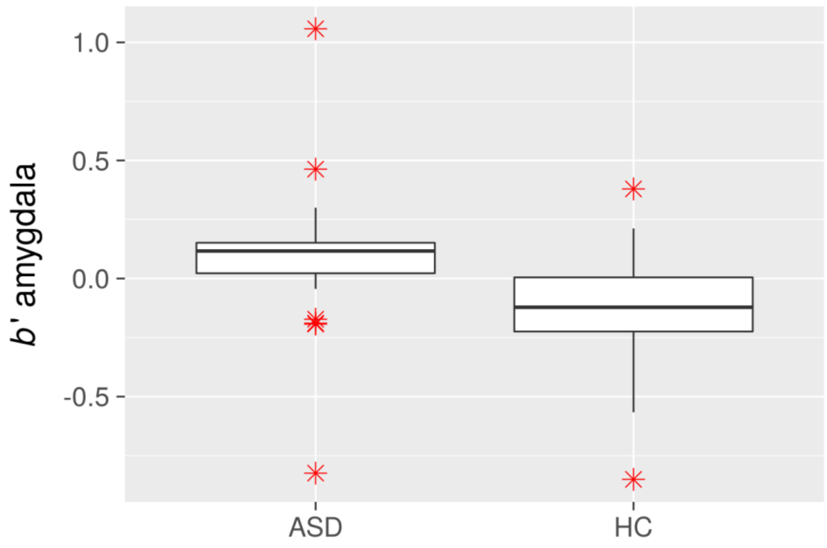

Supplementary Figure S5: Box plot for *b'*amygdala (*b'* for amygdala activation), showing the median, first and third quartile and outliers (asterisks, defined as 1.5 interquartile ranges below the first quartile or above the third quartile). Abbreviations: ASD=autism spectrum disorder, HC=healthy controls.

References

1. Owen, A. M., McMillan, K. M., Laird, A. R. & Bullmore, E. N-back working memory paradigm: A meta-analysis of normative functional neuroimaging studies. *Hum. Brain Mapp.* **25,** 46–59 (2005).
2. Kanwisher, N., McDermott, J. & Chun, M. M. The fusiform face area: a module in human extrastriate cortex specialized for face perception. *J. Neurosci.* **17,** 4302–4311 (1997).
